# Impact of Long-Term Home Quarantine on Mental Health and Physical Activity of People in Shanghai During the COVID-19 Pandemic

**DOI:** 10.3389/fpsyt.2021.782753

**Published:** 2022-01-27

**Authors:** Wentong Zhu, Doudou Xu, Hui Li, Gang Xu, Jingyan Tian, Luheng Lyu, Naifu Wan, Lijiang Wei, Wuwei Rong, Chenchen Liu, Beiwen Wu, Xiaolan Bian, Ankang Lyu

**Affiliations:** ^1^Department of Cardiovascular Medicine, Ruijin Hospital, Shanghai Jiao Tong University School of Medicine, Shanghai, China; ^2^Department of Nursing, School of Medicine, Ruijin Hospital, Shanghai Jiao Tong University, Shanghai, China; ^3^Department of Pharmacy, School of Medicine, Ruijin Hospital, Shanghai Jiaotong University, Shanghai, China; ^4^School of Public Health, School of Medicine, Shanghai Jiao Tong University, Shanghai, China; ^5^Department of Endocrine and Metabolic Diseases, Shanghai Institute of Endocrine and Metabolic Diseases, Ruijin Hospital, Shanghai Jiao Tong University School of Medicine, Shanghai, China; ^6^Shanghai National Clinical Research Center for Metabolic Diseases, Key Laboratory for Endocrine and Metabolic Diseases of the National Health Commission of the PR China, Shanghai Key Laboratory for Endocrine Tumor, State Key Laboratory of Medical Genomics, Ruijin Hospital, Shanghai Jiao Tong University School of Medicine, Shanghai, China; ^7^Department of Biochemistry and Molecular Biology, Miller School of Medicine, University of Miami, Miami, FL, United States

**Keywords:** COVID-19, home quarantine, mental health, physical activity, well-being

## Abstract

This study aimed to investigate the effects of long-term home quarantine on the mental health of people during the COVID-19 epidemic in Shanghai. We conducted an online questionnaire survey on March 26 2020 and collected data on demographics, level of physical activity (PA), and mental health status of the participants. We assessed the mental health status using the Patient Health Questionnaire (PHQ-9) and Generalized Anxiety Disorder Scale (GAD-7), whereas PA was assessed using International Physical Activity Questionnaire Short Form (IPAQ-SF). Of all 2,409 valid samples, participants reported performing a total of 2015.20 metabolic equivalent of task (MET)-minutes/week of total PA before the outbreak period and 1720.29 MET-minutes/week of total PA during the outbreak period (*p* < 0.001). Participants who spent a longer time at home reported to have a better performance on the PHQ-9 (*p* = 0.087) and GAD-7 (*p* < 0.001). A high level of PA was considered an protective factor against depression (OR = 0.755, 95% CI 0.603–0.944, *p* < 0.001). Additionally, a high level of PA had a preventative effect on anxiety (OR = 0.741, 95% CI 0.568–0.967, *p* < 0.001), and a longer working period during the outbreak was shown to be a risk factor for anxiety (11–29 days, OR 1.455, 95% CI 1.110–1.909; 30–60 days OR 1.619, 95% CI 1.227–2.316). Home confinement during the pandemic might not have a negative effect on mental health provided that people engage in more PA indoors. This study encourages interventions for mental health problems through physical exercise.

## Introduction

The Coronavirus Disease 2019 (COVID-19) outbreak started in Wuhan, China in late 2019 and rapidly spread worldwide, resulting in over 140 million infections and 3 million deaths (1) as of April 20, 2021. Mandatory restriction of movement is commonly used to restrain the transmission of infectious diseases, especially respiratory diseases such as the 2003 SARS, MERS, and H1N1 (2). About a year ago, most of China, including Shanghai, adopted strict quarantine measures to control the pandemic. For example, Shanghai launched a Level 1 public health emergency response (3) on January 25, 2020. Facemasks were mandatory in public places, while mandatory temperature screenings were introduced at public places such as hospitals, tourist sites, and commercial centers. Any event that could possibly attract large crowds was banned or delayed. And a large proportion of the population switched to working from home.

China was the first country to bear the brunt of the COVID-19 pandemic, and also one of the first to resume social tranquility. Several studies have focused on psychological health among different groups of people in China. A Chinese survey showed that during the beginning stage of the pandemic, about a third of respondents from the general population suffered from moderate to severe anxiety (4). The burden of psychological stressors on healthcare workers during the epidemic was also a concern (5, 6). Wang et al. conducted a study on the prevalence and associated factors of psychological disorders of the COVID-19 epidemic in China (7, 8). Studies in other countries have also investigated the psychological impact of the COVID-19 pandemic on the general population (9–11) and students (12–16). The results of these studies confirmed that the pandemic had a severe psychological impact on people.

The current study mainly aims to investigate how a change in lifestyle affected mental health during the outbreak period. In this study, we define the 60-day Level 1 public health emergency response declared by Shanghai between January 24 and March 24 as the outbreak period. During the outbreak period, people spent most of their time being quarantined at home, which may increase mental health issues. Mental stressors may include changes in employment, reduced levels of physical activity (PA), a change in working environment, being unable to leave the house and interacting with the outside world, economic adversity due to loss of income, and fear of the COVID-19 pandemic.

The efficacy of exercise as a treatment approach for depression has been demonstrated in several studies. Previous studies have clarified dysregulated pathways as major factors in depression, which include neurotransmitter imbalances, dysregulated inflammatory pathways, HPA disturbances, neuroprogression, increased oxidative stress, and mitochondrial disturbances (17–23). Physical exercise can relieve depression by affecting the pathways mentioned above (24). Moreover, PA has been mentioned as a potential treatment for anxiety (25, 26). In the current study, we focused on how home confinement affects the mental health of workers and its relationship with PA.

As of November 12^th^ 2021, a total of more than 7.1 billion vaccine doses have been administered globally, meanwhile, over 3 million newly confirmed cases were reported in the last 7 days (27). The data revealed vaccination alone is not almighty to beat COVID-19. Other prevention measurements including wearing face masks, keeping social distance and isolation are as crucial. Nie et al. (28). identified long-term home quarantine as one of major factors affecting mental health of Chinese residents and physical exercise was associated with improvement of mental health burden. Faulkner et al. (29). demonstrated that a negative change in exercise behavior during the COVID-19 restrictions was associated with poorer mental health of adults in the UK, Ireland, New Zealand and Australia. The strength of this study is that we provide a novel perspective for people under quarantine, that indoor and outdoor physical exercise is recommended and necessary to improve mental health status. In our study, time duration of home confinement is a key element. During the 60-day outbreak period, working from home became common. On the basis of days at work during the outbreak, we divided the participants into three groups (Table 1). We assumed people who spent different time on home confinement would perform differently on mental health and PA status.

**Table 1:** Demographic characteristics of the participant population, *n* = 2,409 (*n*, %).

**Age (y)**	**37.7 ±9.1**	**Gender**	
≤ 30	599 (24.9)	Male	955 (39.6)
31–40	933 (38.7)	Female	1,454 (60.4)
>40	877 (36.4)	**Education**	
**Days at work during the outbreak Period (d)**	20.5 ± 16.8	High school	214 (8.9)
≤ 10	853 (35.4)	Vocational	320 (13.3)
11–29	715 (29.7)	Undergraduate	1,579 (65.5)
30–60	841(34.9)	Graduate	296 (12.3)
**Weight change**	0.8 ± 2.0	**History of chronic diseases**	
N/A	365 (15.2)	Yes	346 (14.4)
weight unchanged	1,479 (61.4)	No	2,063 (85.6)
weight gained	795 (33)		
weight lost	135 (5.6)		

## Methods

### Study Population

Employed individuals who underwent routine health checkups at Ruijin Hospital were recruited to complete an online questionnaire. A total of 2,580 participants completed the questionnaire, which yielded 2409 samples after data validation.

### Data Collection

A standard questionnaire was designed to obtain participants' demographic information, the number of days they were working during the COVID-19 pandemic (in Shanghai), change in body weight, physical activity intensity, chronic disease history (hypertension, diabetes, coronary artery disease, thrombosis disease, chronic respiratory disease, pulmonary hypertension, liver cirrhosis, chronic kidney disease, chronic gastritis, tumor, etc.), and the state of their mental health (depression and anxiety index).

### Survey Questionnaires

With regard to data privacy and consent for participation, a consent file was obtained prior to completing the questionnaire. Before completing the survey, participants were made aware of their participation in this study. The survey was not anonymous. However, all data collected would only be used for research purposes.

Our team designed an online survey to assess changes in health during the COVID-19 outbreak. In our final survey, we included two questionnaires that evaluate mental health and one that evaluated PA—Patient Health Questionnaire (PHQ-9) (30), Generalized Anxiety Disorder Scale (GAD-7) (31) and International Physical Activity Questionnaire Short Form (IPAQ-SF) (32). Specifically, the participants were told to provide the answers to their IPAQ-SF before and during the outbreak. The entire questionnaire was in Chinese and was available online on March 26, 2020.

#### PHQ-9

The PHQ-9 is a self-reporting diagnostic tool for depression that contains nine items associated with depression-related symptoms (30). Each item is rated as 0 (not at all), 1 (for several days), 2 (at least half of the time), and 3 (nearly every day). A total score of 0–4 points indicates no depressive symptoms, a total score of 5–9 points indicates mild depression, a total score of 10–14 points indicates moderate depression, a total score of 15–19 points indicates severe depression, and a total score of 20–27 points indicates extremely severe depression. The PHQ-9 has been extensively validated and has satisfactory reliability (sensitivity, 0.77; specificity, 0.94) (33). This scale has also been widely used with Chinese populations and has demonstrated excellent psychometric properties (34).

#### GAD-7

The GAD-7 is a seven-item self-reporting scale used to measure generalized anxiety disorder (31). Each item is rated from 0 to 3, similar to PHQ-9 (as described above). Participants who scored ≥5 were considered to be suffering from anxiety. The validity and reliability of the GAD-7 scale in the general population has been confirmed in previous studies (35), and has been widely used in China. Good reliability and validity of the Chinese version of GAD-7 has been confirmed (36).

#### IPAQ-SF

Time data measured by min/week collected from the IPAQ-SF were categorized into different levels of exercise (vigorous, moderate, and walking). METs were matched with each level according to the official IPAQ guidelines: vigorous PA = 8.0 METs, moderate PA = 4.0 METs, and walking = 3.3 METs. According to the IPAQ scoring guide (available at www.ipaq.ki.se), we divided our participants into high, moderate, and low levels of PA. The Chinese version of IPAQ-SF was proved reliable (37).

#### Statistics

The results in Table 2 were presented as mean ± SEM. Comparisons between the two groups were made using the Student *t* test. The positive rates of IPAQ-SF, PHQ-9, and GAD-7 among different working-day groups during the outbreak were compared through χ^2^ tests. A *P* value lower than 0.05 was considered a statistically significant difference. Binary logistic regression models were used to evaluate the association between different factors with PHQ-9 and GAD-7. Statistical analyses were performed with GraphPad Prism 8 for macOS (Graph Pad Prism Software Inc., San Diego, CA, U.S.) and SPSS 25 (SPSS, Inc, Chicago, IL, U.S.).

**Table 2:** IPAQ-SF responses before and during the outbreak.

		**During the outbreak**	**Before the outbreak**	**Δ(%)**	***p* Value**
All PA	MET values	1,720.29 ± 1,813.79	2,015.20 ± 2,100.60	294.91(17.1)	<0.001
Vigorous–intensity activities	Days/week	1.09 ± 1.80	1.26 ± 1.86	0.17 (15.6)	<0.001
	min/week	20.70 ± 32.20	24.30 ± 24.64	3.60 (17.4)	<0.001
	MET values	463.93 ± 1,016.53	543.85 ± 1,020.04	79.92 (17.2)	0.006
Moderate–intensity activities	Days/week	2.74 ± 2.64	2.77 ± 2.61	0.03 (1.1)	0.629
	min/week	45.43 ± 47.29	44.90 ± 45.15	0.53 (1.2)	0.692
	MET values	750.93 ± 1,098.53	714.50 ± 1,005.96	36.43 (5.1)	0.230
Walking	Days/week	2.87 ± 2.57	3.96 ± 2.59	1.09 (38.0)	<0.001
	min/week	38.85 ± 39.05	47.96 ± 41.80	9.11 (23.4)	<0.001
	MET values	505.43 ± 684.32	756.84 ± 816.94	251.41 (49.7)	<0.001
Sitting	Hours/day	6.13 ± 3.20	5.66 ± 3.15	0.47 (8.2)	<0.001

## Results

### Participant Characteristics

A total of 2,580 participants completed the online survey between March 26 and May 9, 2020, which yielded 2,409 valid samples. The mean age of respondents was 37.7 years (range: 20–88). 39.6% of the participants were male, and 77.8% possessed a high level of education (undergraduate and above). 14.4% of the participants had a history of chronic disease, 33% gained weight during the outbreak period, and 5.6% reported losing weight. We divided the entire data sample by the number of days worked at home during the outbreak period. Out of the 2,409 participants, 853 (35.4%) worked for <10 days, 715 (29.7%) worked between 11 and 29 days, and 841 people worked for more than 30 days.

### Physical Activity Before and During the Outbreak Period

As shown in Table 1, the average weight change was positive, with 33% of the participants reporting weight gain during the outbreak. We compared the responses to the PA questionnaire (IPAQ-SF) recorded before and during the outbreak period, and the results are presented in Table 2.

Participants reported performing a total of 2015.20 MET-minutes/week of total PA before the outbreak period, and 1720.29 MET-minutes/week of total PA during the outbreak period (*p* < 0.001). The number of days/week and minutes/day of vigorous intensity PA during the outbreak decreased by 15.6% (*p* < 0.001) and 17.4% (*p* < 0.001), respectively. In addition, the MET values of vigorous-intensity PA were 17.2% lower than those before the outbreak period (*p* = 0.006). The number of days per week of moderate intensity PA decreased by 1.1% during the outbreak period (*p* = 0.629), whereas the amount of minutes/day of moderate intensity PA increased by 1.2% during the same period (*p* = 0.692). Additionally, the MET values of moderate intensity PA were 5.1% higher during the outbreak period (*p* = 0.230). The number of days/week of walking reduced by 38% during the outbreak period (*p* < 0.001). Likewise, the amount of minutes/day of walking reduced by 23.4% during the outbreak period (*p* < 0.001). Additionally, MET values of walking were also revealed to be 49.7% lower during the outbreak period (*p* < 0.001). Statistical analysis also revealed that the amount of hours/day of sitting increased by 8.2% during the outbreak period (*p* < 0.001).

### PA Intensity, Depression, and Anxiety Proportions in Different Lengths of Home Confinement

We used the Chi-square test to further investigate the relationship between the length of time spent working during the outbreak period, PA levels, and mental health status (Table 3). We found PA levels (*p* < 0.001), GAD-7 score (*p* < 0.001), and PHQ-9 score (*p* = 0.087) to be associated with different working times during the outbreak period.

**Table 3:** Comparison on the positive rates of IPAQ-SF, PHQ-9, and GAD-7 among different working-day groups during the outbreak.

**Surveys**	**30–60d**	**11–29d**	**0–10d**	**χ^2^**	***p*-value**
PA level (High/Total)	199/841	155/715	266/853	21.298	<0.001
GAD-7 ≥5/Total	149/841	138/715	107/853	14.732	<0.001
PHQ-9 ≥5/Total	217/841	187/715	187/853	4.886	0.087

### Average PHQ-9/GAD-7 Scores in Different Characteristic Groups of Participants

**Figure 1:**
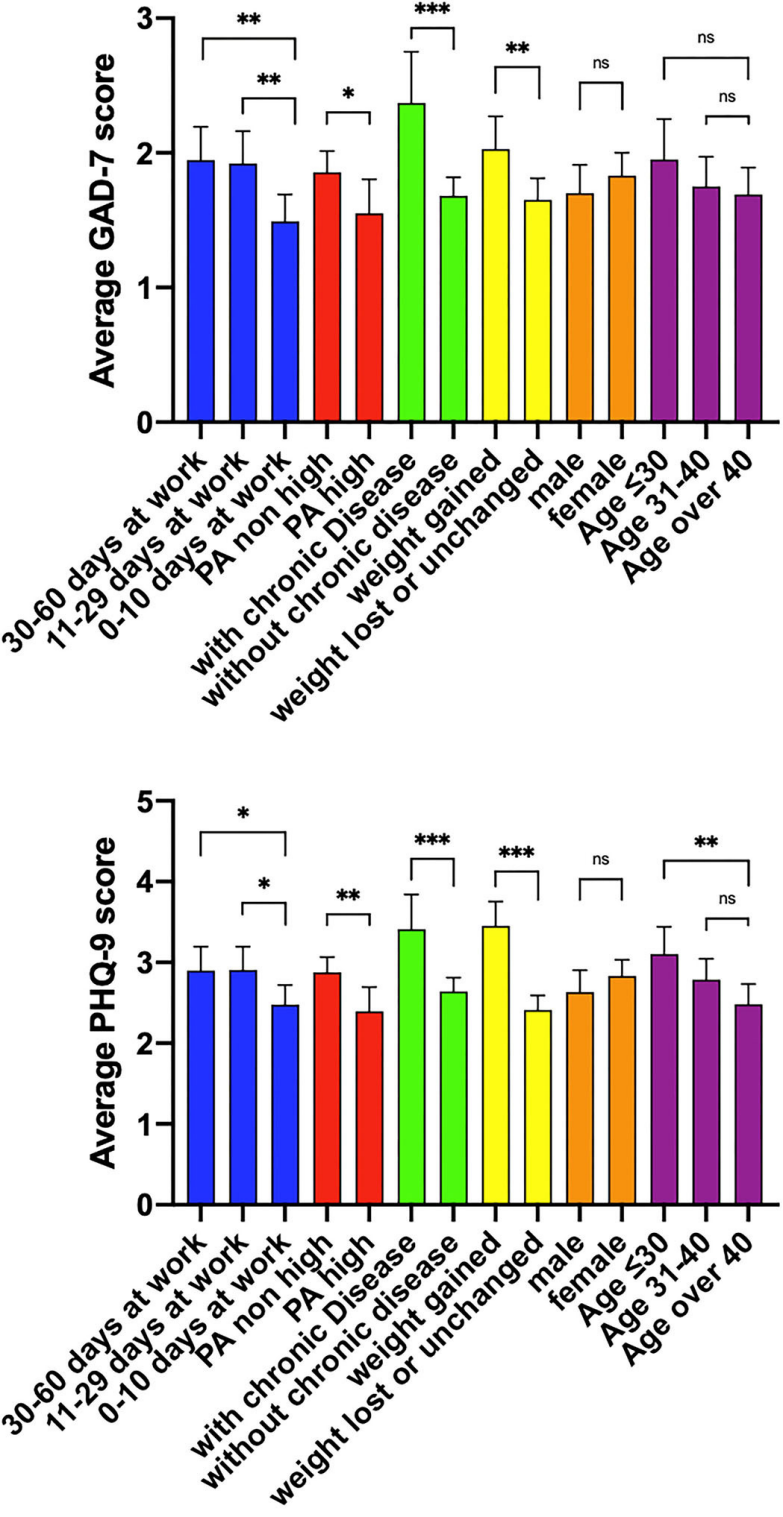
Average GAD-7/PHQ-9 scores in different characteristic groups of participants. PA high: participants who had high level of physical activity. PA non-high: participants who had medium or low level of physical activity. According to the IPAQ scoring guide listed previously, Physical activity status of the participants were graded high, medium and low. In this article, medium and low levels of physical activity were combined as PA non-high. The other characteristic groups were described in Table 1. ^*^p < 0.05, ^**^p < 0.01, ^***^p < 0.001, ns, not significant.

As shown in Figure 1, people who worked 30–60 days and 11–29 days during the outbreak period reported significantly higher average GAD-7 scores than those who worked for <10 days (*p* < 0.01). People who maintained a high level of PA intensity scored significantly lower than those who reported moderate and low levels of PA intensity (*p* < 0.05). Participants with a history of chronic diseases scored notably higher than those without (*p* < 0.001). People who gained weight during the outbreak period also reported significantly higher scores than those who did not gain weight (*p* < 0.01). However, gender and age groups did not show any statistical significance on their performance on the GAD-7 scale.

In the case of PHQ-9 scores, people who went to work for 30–60 days and 11–29 days during the outbreak period reported significantly higher average GAD-7 scores than those who went to work for <10 days (*p* < 0.05). People who maintained a high level of PA intensity scored significantly lower than those who reported moderate or low levels of PA intensity (*p* < 0.01). Participants with a history of chronic diseases scored notably higher than those who did not (*p* < 0.001). People who gained weight during the outbreak period showed significantly higher scores than those who did not gain weight (*p* < 0.001). Similar to the GAD-7 results, gender differences in PHQ-9 performance were also minor. For the age groups, individuals younger than 30 years scored significantly higher than those aged >40 years (*p* < 0.01).

### Binary Logistic Regression Analysis of PHQ-9 and GAD-7 Scores

#### Binary Regression Model for PHQ-9

As shown in Table 4, the relatively older participants tended to perform better in the PHQ-9 survey than the younger ones (OR 0.978, 95% CI 0.967–0.988), suggesting that age could be a protective factor. A higher PA level was also seen to be a protective factor (OR 0.755, 95% CI 0.603–0.944). Gaining weight during the outbreak (OR 1.754, 95% CI 1.466–2.217) and a history of chronic diseases (OR 1.711, 95% CI 1.312–2.233) were risk factors for depression.

**Table 4:** Regression results for the PHQ-9 and GAD-7 scores.

						**95% Confidence interval for exp (B)**	
	**B**	**SE**	**Wald**	**Sig**	**Exp (B)**	**Lower bound**	**Upper bound**
**PHQ-9**							
Age	−0.23	0.006	16.19	<0.001	0.978	0.967	0.988
Gaining weight	0.562	0.098	32.574	<0.001	1.754	1.446	2.127
History of chronic diseases	0.537	0.136	15.678	<0.001	1.711	1.312	2.233
High-level PA during outbreak	−0.282	0.114	6.071	0.014	0.755	0.603	0.944
**GAD-7**							
Gaining weight	0.281	0.115	5.955	0.015	1.324	1.057	1.659
History of chronic diseases	0.561	0.141	15.8	<0.001	1.752	1.329	2.311
High-level PA during outbreak	−0.300	0.136	4.868	0.027	0.741	0.568	0.967
**Days at work**							
0–10 days^*^			12.951	0.002			
11–29 days	0.375	0.138	7.359	0.007	1.455	1.11	1.909
30–60 days	0.482	0.141	11.587	0.001	1.619	1.227	2.136

*Parameter estimates for predictors in each logistic regression model*.

* *means reference*.

#### Binary Regression Model for GAD-7

Similarly, gaining weight during the outbreak (OR 1.324, 95% CI 1.057–1.659) and a history of chronic diseases (OR 1.752, 95% CI 1.329–2.311) were risk factors for anxiety. Higher PA level was also seen to be a protective factor (OR 0.741, 95% CI, 0.568–0.967). Compared to participants who worked less than 10 days during the outbreak, participants who spent 11-29 days (OR 1.455, 95% CI 1.110–1.909) and more than 30 days at work (OR 1.619, 95% CI 1.227–2.316) were more likely to score over 5 in the GAD-7 survey, which indicates that going to work was a huge risk factor during the outbreak (Table 4).

## Discussion

Home confinement due to the current COVID-19 pandemic has dramatically impacted lifestyle activities globally, especially in terms of PA (38, 39). Overall, we found that differences in the length of home confinement during COVID-19 can have different levels of influence on mental health. Before the survey data was analyzed, we presumed that a longer time spent in quarantine might have had an adverse impact on mental health, and that not being able to socialize could be a significant source of psychological stress (40). Our presumption is supported by a study focusing on psychological distress during the SARS epidemic reported that symptoms of PTSD and depression increased by 28.9 and 31.2%, respectively. A longer duration of quarantine was associated with the increased prevalence of PTSD symptoms (41).

Interestingly, inconsistent with the previous studies mentioned above, our study showed that individuals who spent a longer time at home were more likely to have higher levels of PA and performed better in the PHQ-9 and GAD-7 surveys. The reasons behind this may include the following: going outdoors meant being exposed to more risk of contact with the virus than staying at home, and staying indoors would give people more time to spend on PA.

In this study, we found that the intensity of PA during the outbreak period was significantly lower than that before (*p* < 0.001, Table 2), and this finding was supported by a previous international study (42). The most significant change was in walking, which decreased by nearly 50% in MET value during the outbreak period. A 17.2% reduction in MET values of vigorous activity and 8.2% increase in hours of sitting were also notable. However, the intensity of moderate PA during and before the outbreak period were similar. According to the results of this study, the walking capacity was significantly reduced due to confinement. Hence, we encourage diversified indoor sports activities as an alternative. We also noted that regular PA may play an important role in relieving the symptoms of anxiety and depression. Although there is no evidence that PA can prevent the onset of depression, exercise can reduce the possibility of aggravating the symptoms in patients with mild depression (43) given that depression is commonly associated with low levels of PA. One study on data from over 4,000 adults showed that people with depression spent significantly less time doing light and moderate PA (44). In addition to depression, the protective effect of PA on generalized anxiety disorder has also been proven in another study. The odds of developing GAD was reduced by approximately 57% among older adults who met WHO PA guidelines (45). A cross-sectional study of 1.2 million people reported that regular PA has a positive effect on mental health (46).

According to previous studies, the 1-month prevalence of a major depressive disorder was 5.2% in a sample representing the general population (47). Accordingly, the cut-off PHQ-9 score was set at five in the current study. Based on our cut-off score, 591 participants out of 2,409 (24.5%) were considered to have mild depression or above. If we adjust the cut-off score to 10 points, 138 participants (5.7%) would be considered to have moderate or high levels of depression, consistent with the prevalence from the study mentioned above. Generalized anxiety disorder has an estimated prevalence in the general population of 1.6% to 6.2% (48–51). Among the 2,409 participants in the current study, 394 (16.4%) scored more than five points, and 81 (3.4%) scored more than 10 points in the GAD-7 survey. Previous studies showed that GAD-7 mean scores of the sample representing the general population ranged from 2.0 points (52) to 8.0 points (53). In our study, the mean scores of our samples was 1.8%.

Since the beginning of the 21^st^ century, humankind have suffered subsequently from the SARS in 2003, H1N1 in 2009, MERS in 2012, Ebola virus disease (EVD) in 2014, and the new COVID-19 in 2019, five public health emergencies caused by infectious disease. Problem of mental health crisis has gained increasing attention. There's no doubt that eliminating the existence of the disease is the best way to avoid public mental health crisis (54). Sports was considered to be effective to promote mental health (55). Previous researchers have suggested various mechanisms of positive effect of physical activity on mental health (56, 57). What kind of types of physical activities are more accessible and practical for people especially under confinement? Future studies are needed to explain how to maintain physical activity during a global health crisis. To explore effectiveness and efficiency of physical activity to intervene impaired mental health, cross-sectional, multicenter studies of large sample sizes should be encouraged.

This study also showed that individuals who spent longer time at home during the outbreak period were more likely to have higher levels of PA, and they performed better in the PHQ-9 and GAD-7 surveys. A reasonable explanation for this interesting result could be as follows: At the initial stage of the COVID-19 epidemic, outdoor activity carried a higher risk of exposure to the virus. More people started working from home for a longer period of time, which gave them more time to exercise freely.

## Conclusion

To our knowledge, our study is the first to focus on a unique demographic of people undergoing health checkups, a demographic that is characterized by a stable income and a relatively high level of education. This study provided valuable information to people suffering from home confinement. We found that home confinement during a pandemic is not detrimental to mental health provided that people engage in more PA indoors. Therefore, we encourage people who are being quarantined to spend more time doing physical exercise to reduce the risk of developing depression, generalized anxiety disorder, or any other potential mental health issues.

The present study has several limitations. First, the data collected were based on an online survey, which required the participants to assess their levels of PA prior to the pandemic. It was unrealistic to design a prospective study in response to the current pandemic. Second, the time frame for the current study was only 60 days. As the pandemic develops further, the relationships between the measures and various factors in the study might change. Future research should include larger population samples to further confirm the current findings.

## Data Availability Statement

The raw data supporting the conclusions of this article will be made available by the authors, without undue reservation.

## Ethics Statement

The studies involving human participants were reviewed and approved by Ruijin Hospital Ethics Committee, Shanghai Jiao Tong University School of Medicine. The patients/participants provided their written informed consent to participate in this study.

## Author Contributions

AL, JT, BW, GX, and XB: designed the study. AL, JT, BW, WZ, NW, and LL: designed the questionnaire. WZ, LW, WR, and CL: recruited participants and collected data. WZ, DX, HL, and GX: performed the statistical analysis. WZ: wrote the first draft. All authors revised, read, and approved the final manuscript.

## Funding

The study was funded by the National Natural Science Foundation of China (grant number 81770051 and 81930002).

## Conflict of Interest

The authors declare that the research was conducted in the absence of any commercial or financial relationships that could be construed as a potential conflict of interest.

## Publisher's Note

All claims expressed in this article are solely those of the authors and do not necessarily represent those of their affiliated organizations, or those of the publisher, the editors and the reviewers. Any product that may be evaluated in this article, or claim that may be made by its manufacturer, is not guaranteed or endorsed by the publisher.
